# Single‐Cell RNA Sequencing Revealed the Role of Interferon‐Gamma Related Genes in Primary Sjögren's Syndrome

**DOI:** 10.1111/jcmm.71313

**Published:** 2026-08-07

**Authors:** Lei Shi, Tian‐chi Wei, Jing Zhang

**Affiliations:** ^1^ Department of Rheumatology and Immunology The Second Hospital of Shanxi Medical University Taiyuan China; ^2^ Shanxi Medical University Taiyuan China; ^3^ Department of Clinical Laboratory The Second Hospital of Shanxi Medical University Taiyuan China

**Keywords:** diagnostic model, interferon‐gamma, macrophage heterogeneity, primary Sjögren's syndrome, single‐cell RNA sequencing

## Abstract

Primary Sjögren's syndrome (pSS) is a chronic autoimmune disorder. Dysregulated interferon‐gamma (IFN‐γ) signalling is implicated in pSS pathogenesis, yet the underlying mechanisms remain elusive. This study aimed to identify key IFN‐γ‐associated diagnostic genes and delineate their roles in immune dysregulation using peripheral blood transcriptomic and single‐cell RNA sequencing (scRNA‐seq) data. Publicly available Gene Expression Omnibus (GEO) datasets were used, and the bioinformatics findings were further supported by reverse transcription‐quantitative PCR (RT‐qPCR) validation. Peripheral blood transcriptomic data from training and validation cohorts were analysed, and key genes were screened using machine learning algorithms, receiver operating characteristic (ROC) curve analysis, and differential expression analysis. A diagnostic model and nomogram were subsequently constructed. Immune infiltration, pathway enrichment, and gene regulatory networks were also investigated, while scRNA‐seq analysis was performed to characterize cellular heterogeneity, pseudotime trajectories, and cell–cell communication. Four key genes, HERC6, IL15, CD58 and PTGS2, were identified, and the resulting diagnostic model and nomogram demonstrated high diagnostic accuracy. Immune profiling revealed marked dysregulation of the immune microenvironment in pSS, accompanied by extensive alterations in metabolic and signalling pathways. Pathway enrichment analysis further demonstrated both distinct and shared functional roles of the four key genes. Single‐cell analysis suggested that macrophages may act as central regulators in pSS pathogenesis, as evidenced by altered pseudotime trajectories, enrichment in early differentiation states, and intensified cell–cell communication networks. RT‐qPCR further confirmed the significant upregulation of HERC6, IL15, and PTGS2 in patients with pSS. Collectively, this study established an IFN‐γ‐associated diagnostic model for pSS and implicated macrophages as potential key contributors to immune dysregulation through altered differentiation, enhanced intercellular communication, and IFN‐γ‐associated gene expression. These findings provide new insights into the pathogenesis of pSS and identify potential diagnostic biomarkers and therapeutic targets.

## 1 Introduction

In Sjögren's Syndrome (SS), a chronic autoimmune disease, exocrine glands (notably the salivary and lacrimal glands) undergo lymphocytic infiltration, which is the disease's defining characteristic. This leads to progressive glandular dysfunction, resulting in xerostomia and xerophthalmia [1]. Clinically, it frequently involves multiple organ systems. Dry skin and pruritus are the most common dermatological manifestations, alongside leukocytoclastic vasculitis, subacute cutaneous lupus erythematosus‐like skin lesions, myalgia, arthralgia and neuropsychiatric symptoms [2]. Regarding the pathogenesis of Primary Sjögren's syndrome (pSS), multiple hypotheses exist, with a prevailing view suggesting disorders in both innate and adaptable immunity. Notably, dysregulation at the interface between immune system abnormalities and neuroendocrine system is considered pivotal in tear and salivary gland dysfunction [3]. Currently, the diagnosis of pSS relies on a combination of clinical, histological, and immunological findings, as well as related subjective symptoms (dry eye and/or dry mouth) [4]. Existing topical treatments (such as artificial tears and saliva substitutes) can only alleviate symptoms and are unable to reverse glandular atrophy or restore secretory function [5]. The traditional immunomodulator hydroxychloroquine (HCQ) provides only mild relief for pain, and there is a paucity of large‐scale clinical evidence supporting its efficacy [6]. Therefore, it is essential to further elucidate the key pathogenic pathways in pSS and explore more specific diagnostic biomarkers and potential therapeutic targets, thereby providing a theoretical basis for optimizing the clinical diagnosis and treatment strategies for pSS.

Interferon‐gamma (IFN‐γ), a key soluble cytokine produced by T lymphocytes, macrophages, mucosal epithelial cells, and natural killer cells, plays a central role in both innate and adaptive immunity [7, 8]. By binding to IFN‐γ receptors, it activates the Janus kinase/signal transducer and activator of transcription (JAK/STAT) signalling pathway, thereby inducing the expression of IFN‐γ‐stimulated genes [7, 9]. Abnormal IFN‐γ expression and function have been implicated in numerous human diseases [10]. The role of IFN‐γ has garnered increasing attention in research on the pathogenesis of SS. Evidence indicates that in SS, IFN‐γ triggers ferroptosis in salivary gland epithelial cells (SGECs) via Janus kinase/signal transducer and activator of transcription 1 (JAK/STAT1)‐mediated cystine‐glutamate exchanger (system Xc‐) inhibition [11]. Furthermore, studies have revealed that IFN‐γ secreted by CD4+ T cells in SS patients induces ferroptosis in SGECs and suppresses aquaporin 5 (AQP5) expression [12]. TH1 cells producing IFN‐γ and dysfunctional regulatory T cells are believed to jointly participate in the pathogenesis of SS [13]. Therefore, in‐depth investigation of the expression characteristics of IFN‐γ in SS and its immune cell sources is crucial for comprehensively understanding the immunopathological mechanisms of this disease. It may also provide new insights for the discovery of clinical diagnostic markers and the development of personalized therapeutic targets.

Abnormal transcriptional and post‐transcriptional regulation also plays a key role in immune dysregulation in pSS. Multiple studies have shown that dysregulated miRNAs contribute to pSS pathogenesis by modulating genes and pathways involved in inflammatory and interferon‐associated responses, while disease‐related miRNA expression signatures may have potential as candidate diagnostic biomarkers [14, 15, 16]. In parallel, dysregulation of the interferon signalling axis mediated by transcription factors (TFs) has also been confirmed to be closely associated with glandular damage. However, constructing a TF‐miRNA‐mRNA regulatory network based on IFN‐γ signalling for pSS and analysing its cell‐specific mechanisms using single‐cell data remains rarely reported. However, these molecular mechanisms remain incompletely characterized at the cellular resolution.

In recent years, the rapid advancement of single‐cell RNA sequencing (scRNA‐seq) technology has significantly advanced research in immunology, particularly in deciphering the high cellular heterogeneity of immune cells. This technology enables precise characterization of the functional states and transcriptional profiles of various immune cells within distinct immune microenvironments [17]. For immune‐mediated diseases such as SS, scRNA‐seq provides unprecedented resolution at the cellular level [18, 19]. By leveraging this technique, researchers can accurately identify key immune cell subsets secreting IFN‐γ and their activation status in SS patients, thereby offering deeper glimpses into the specific roles of particular immune cells in the pathogenesis and progression of the disease.

This study conducted bioinformatic analysis based on public transcriptome and single‐cell sequencing data from the Gene Expression Omnibus (GEO) database, representing a retrospective secondary data analysis, with preliminary experimental validation via reverse transcription‐quantitative PCR (RT‐qPCR). All transcriptome and single‐cell sequencing data were derived from peripheral blood of patients with pSS and healthy controls, and the relevant results should be interpreted in conjunction with the immune characteristics of peripheral blood. The research aimed to integrate scRNA‐seq technology to untangle the key genes regulated by IFN‐γ and their associated immune response pathways, and to further elucidate the IFN‐γ‐related immune cell populations and their functional states in patients with SS. Thereby providing novel insights into the pathophysiology of SS.

## 2 Materials and Methods

### 2.1 Data Acquisition

The data for this study were gained from the GEO database, with the accession number GSE84844 (using the GPL570 platform). This dataset contained whole blood expression profiles from 30 pSS patients and 30 healthy volunteers. The validation was downloaded from the GEO database (accession GSE48378, GPL5175 platform), comprising peripheral blood samples from 11 pSS patients and 16 healthy controls. Raw data for both datasets were downloaded on July 10, 2025. The scRNA‐seq dataset was also retrieved from the GEO database (accession GSE157278, GPL24676 platform) on July 14, 2025. It included peripheral blood mononuclear cells (PBMCs) from 5 pSS patients and 5 healthy controls. In addition, a non‐redundant set of 288 IFN‐γ response genes (IFNG‐RGs) was curated from the ‘HALLMARK INTERFERON GAMMA RESPONSE’ (*n* = 200) and ‘GOBP RESPONSE TO TYPE II INTERFERON’ (*n* = 88) gene sets in the Molecular Signatures Database (MSigDB) on July 10, 2025, with redundant entries removed (Supplementary Table 1).

### 2.2 Identification of IFNG Associated Candidate Genes

Differentially expressed genes (DEGs) between pSS patients and healthy controls in the training cohort were recognized via the R package ‘limma’ (v 3.56.2) [20]. Benjamini–Hochberg (BH) correction was applied to control false discovery rate (FDR), with DEGs defined as genes meeting |log_2_FC| > 0.5 and adjusted *p* < 0.05. A volcano plot was generated to depict the distribution of DEGs. Expression patterns of the top 10 up‐/down‐regulated DEGs were illustrated via a heatmap using the R package ‘ComplexHeatmap’ (v 2.16.0) [21]. To delineate genes concurrently linked to pSS pathogenesis and IFN‐γ response, a Venn diagram was constructed by the R package ‘ggvenn’ (v 0.1.10) to converge the DEGs with the IFNG‐RGs [22]. The overlapping genes were designated as candidate genes.

### 2.3 Functional Enrichment and Protein Interaction Analysis

To investigate the biological functions and pathways associated with the candidate genes, Gene Ontology (GO) and Kyoto Encyclopedia of Genes and Genomes (KEGG) enrichment analyses were carried out using the R package ‘clusterProfiler’ (v 4.8.1) [23]. Gene symbols were mapped to Entrez IDs via clusterProfiler's bitr; GO/KEGG enrichment used org.Hs.eg.db full human genome as background. GO analysis covered three ontologies, including biological process (BP), cellular component (CC), and molecular function (MF). Enriched terms within each ontology were selected based on a significance threshold of adjusted *p*‐value (BH false discovery rate, FDR) < 0.05. KEGG pathway enrichment analysis was conducted using the same significance threshold (adjusted *p* < 0.05). Protein–protein interaction (PPI) networks were constructed using the Search Tool for the Retrieval of Interacting Genes/Proteins (STRING) database, accessed on July 14, 2025. STRING interactions with confidence ≥ 0.9 were kept using all evidence sources; nodes with degree ≥ 1 were retained, isolated nodes removed. The resulting PPI network was visualized via the R package ‘ggraph’ (v 2.2.1) [24].

### 2.4 Machine Learning (ML) Algorithms

To identify the most relevant feature genes, the Boruta algorithm and least absolute shrinkage and selection operator (LASSO) regression were employed. The Boruta algorithm was performed using the R package ‘Boruta’ (v 8.0.0) [25]. To ensure robustness and prevent over‐retention of features, the maximum number of importance source runs (maxRuns) parameter was set to 100. In each iteration, Boruta compares the importance of original features with that of randomly permuted shadow features, retaining only those that consistently outperform the maximum shadow feature importance across iterations while progressively eliminating irrelevant features. This iterative strategy identifies all potentially relevant features rather than a minimal subset. After multiple iterations converging to a stable solution, some feature genes were identified as potentially relevant and retained. LASSO regression was executed by the R package ‘glmnet’ (v 4.1.8) [26]. By imposing an L1 penalty, LASSO performs simultaneous variable selection and regularization, shrinking some regression coefficients exactly to zero to reduce multicollinearity and overfitting. To guarantee the stability and reliability of the results, a 5‐fold cross‐validation procedure was implemented. The optimal lambda value (lambda.min) was determined based on the criterion of achieving the minimum cross‐validation error. Subsequently, genes with non‐zero coefficients under this optimal lambda were identified. Feature genes were determined by taking the intersection of the genes selected by the Boruta algorithm and those selected by the LASSO regression analysis.

### 2.5 Evaluation of Diagnostic Potential and Expression Consistency

The diagnostic potential of the identified feature genes for distinguishing pSS patients was gauged applying receiver operating characteristic (ROC) curve analysis using the roc() function in the R package ‘pROC’ (v 1.19.0.1) in both the training and validation cohorts. The Area Under the Curve (AUC) and its 95% confidence interval (CI) were calculated as the primary metric to assess the discriminatory ability of individual genes, with an AUC > 0.7 prespecified as indicating acceptable discriminatory performance. Furthermore, to appraise the consistency of expression patterns of these genes within two cohorts, differential expression analysis between pSS patients and healthy controls was executed via the Wilcoxon test (*p* < 0.05). Visualization of expression patterns across groups was achieved.

### 2.6 Logistic Regression Model Construction and Performance Evaluation

A Logistic Regression model was employed for predicting pSS. The performance of the logistic regression model was judged via ROC curve analysis. The ROC curves and corresponding AUC values were computed for both the training and validation cohorts to assess the model's discriminatory ability and generalizability. The ROC analysis was performed using the roc function from the R package ‘pROC’ (v 1.19.0.1) [27]. Model performance was considered robust if the AUC exceeded 0.7 in two cohorts.

### 2.7 Nomogram Construction and Validation

To enhance the clinical interpretability and utility of the predictive model, a nomogram was built based on the key genes. The nomogram was developed by the R package ‘rms’ (v 6.8.1) [28]. A calibration curve was plotted to visually depict the relationship between the nomogram's predicted probabilities and the actual probabilities. Model calibration was further assessed using the Hosmer–Lemeshow goodness‐of‐fit test, with a non‐significant result (*p* > 0.05) indicating adequate fit between predicted and observed probabilities. The Hosmer–Lemeshow test was performed to statistically evaluate the goodness‐of‐fit of the nomogram. A non‐significant result (*p* > 0.05) suggested that the model's predictions do not significantly deviate from the observed outcomes. To assess the clinical net benefit and utility of the nomogram across a range of threshold probabilities, decision curve analysis (DCA) was employed, which quantifies net benefit relative to the ‘treat all’ and ‘treat none’ strategies. The DCA was performed to estimate the potential clinical impact of applying the nomogram for pSS risk prediction.

### 2.8 Immune Infiltration Analysis

To characterize the immune microenvironment differences between pSS patients and healthy controls, the relative abundance of 64 distinct immune cell types was assessed using the R package ‘xCell’ (v 1.1.0) [29]. xCell uses purified cell‐type gene signatures to calculate enrichment scores for 64 immune/stromal cell populations from bulk RNA‐seq. Given peripheral blood input, scores represent blood immune cell proportions. To identify differentially infiltrated immune cells between the two groups, the Wilcoxon test was employed (*p* < 0.05). To investigate the relationships between the identified differentially infiltrated immune cells, pairwise correlation analysis was carried out via the ‘psych’ package (v 2.5.3) [30]. Correlations with an absolute value > 0.3, a *p* < 0.05 were regarded statistically meaningful. The potential associations between the previously identified key genes and the differentially infiltrated immune cells were also explored; the relationships between the overall IFN‐γ response signature and differently infiltrated immune cells were assessed (|cor| > 0.3, *p* < 0.05).

### 2.9 Gene Set Variation Analysis (GSVA) and Gene Set Enrichment Analysis (GSEA)

To elucidate the biological pathway differences between pSS patients and healthy controls, GSVA was employed. Specifically, the GSVA score matrix was generated by the gsva function from the R package ‘GSVA’ (v 1.48.2) [31]. Differential analysis of the resulting GSVA score matrix was then performed using the R package ‘limma’ (v 3.56.2). A contrast matrix was created using the makeContrasts function to define comparisons between pSS and normal groups. Linear models were fitted to the data using the lmFit function, and empirical Bayes testing was applied via the eBayes function to identify differentially enriched pathways (*p* < 0.05, |t| > 2). The top five upregulated and downregulated pathways were extracted for visualization.

To determine the functional associations and regulatory trends of key genes, GSEA was implemented in the training cohort. The KEGG pathway gene set (c2.cp.kegg.v7.4.symbols.gmt) was utilized. Initially, the cor values were calculated between each key gene and all other genes. Genes were then ranked based on the magnitude of these cor values to generate a sorted gene list. This ranked list served as the leading sequence, and GSEA was implemented by the R package ‘clusterProfiler’, along with the KEGG pathway gene set. Enriched pathways were filtered using thresholds of *p* < 0.05, FDR < 0.25, and absolute |NES| > 1. The top five enriched pathways for each key gene were selected for visualization based on *p*‐value ranking.

### 2.10 Gene Interaction Network Analysis

To probe interactions among key genes and their functional roles in biological processes, a gene interaction network analysis was executed via the GeneMANIA tool. The list of key genes was input into the GeneMANIA online interface to construct a functional association network. Furthermore, to investigate the transcriptional and post‐transcriptional regulation of the key genes, a regulatory network analysis was performed using the NetworkAnalyst database. This analysis aimed to identify transcription factors (TFs) targeting the key genes and microRNAs (miRNAs) predicted to regulate their mRNA transcripts. The resulting TF‐mRNA‐miRNA regulatory network was built and illustrated.

### 2.11 Data Preprocessing and Cell Type Annotation

The raw gene expression matrix was utilized to construct a Seurat object using the CreateSeuratObject function within the R package ‘Seurat’ (v 5.3.0) [32]. Rigorous quality control (QC) was applied to exclude potentially low‐quality cells and doublets. Quality control thresholds were applied as follows: genes detected in fewer than 3 cells were excluded; cells expressing fewer than 200 or more than 4000 genes were removed to exclude empty droplets and potential doublets; cells with mitochondrial gene content ≥ 15% or total UMI count ≥ 10,000 were filtered out to remove low‐quality or dying cells. After filtering, 56,012 cells and 21,176 genes were retained from an initial pool of 60,671 cells. The filtered expression data were standardized via the NormalizeData function from the R package ‘Seurat’ (v 5.3.0). Highly variable genes (HVGs) were identified. The LabelPoints function was used to visualize the top 10 most variable genes. Following the identification of HVGs, the expression data for all genes across all cells were scaled using the Scale Data function in the ‘Seurat’ package. Principal component analysis (PCA) was then executed on the scaled HVGs to assess the overall distribution of cells across samples and identify potential outlier samples. The number of principal components (PCs) used for downstream analysis was determined by both ElbowPlot and JackStraw permutation analysis; PCs 1–20 were selected as the elbow plot showed a plateau beyond PC 20 and JackStraw analysis indicated significant deviation from the null distribution (*p* < 0.05). Cell clustering was performed on the cells embedded within the determined number of PCs via the *t*‐distributed stochastic neighbour embedding (*t*‐SNE). Clustering resolution was set to 0.3. The clusters identified by t‐SNE were manually annotated to define major cell types. Annotation was performed by integrating marker gene expression information derived from two sources: (1) cell type‐specific marker genes reported in the reference literature [33], and (2) marker genes for corresponding cell subpopulations retrieved from the CellMarker 2.0.

### 2.12 Analysis of Cell Type Differences and Gene Expression

To investigate cellular heterogeneity between pSS patients and healthy controls, differential analysis was performed to compare cell counts or percentages between pSS patients and healthy controls using the Wilcoxon test, with statistical significance assessed at a threshold of *p* < 0.05. Differences in cell type proportions were additionally evaluated using the chi‐squared test (*p* < 0.05) to test whether observed distributions deviated from expected proportions under the null hypothesis. Subsequently, HVGs were identified for each cell type. The top 5 HVGs were selected based on their variability scores, and their expression levels were visualized. Following this, key genes were analysed for their expression patterns across different cell types. The average expression proportion of key genes was calculated for each cell type. The cell type exhibiting the highest average expression proportion of key genes was designated as the key cell type. Finally, the expression distribution of key genes was visualized using t‐SNE plots.

### 2.13 Differential Gene Expression of Key Cell Type and Functional Enrichment

To probe the functional roles of key cell types in pSS pathogenesis, single‐cell transcriptomic data were analysed via the R package ‘Seurat’. DEGs between pSS patients and healthy controls were identified using the FindMarkers() function with default Wilcoxon test parameters set as logfc.threshold = 0.25 and min.pct = 0.25. Within key cell clusters, differential expression was assessed using the Wilcoxon rank‐sum test as implemented in FindMarkers(). Genes with adjusted *p* < 0.05 (BH correction) were considered significant. Genes were ranked by adjusted *p*‐value and average expression difference, and those with an adjusted *p* < 0.05 were retained as significant scRNA‐seq DEGs. For functional annotation, GO enrichment analysis and KEGG pathway analysis were performed using the R package ‘clusterProfiler’ with the ‘org.Hs.eg.db’ annotation database. Enriched terms were sorted by adjusted *p*‐value, and the top 5 substantial GO terms and KEGG pathways were visualized.

### 2.14 Pseudotime Trajectory Analysis

Developmental dynamics of key cell types were inferred using the R package ‘monocle’ (v 2.30.1) [34]. Cells were re‐clustered into some subpopulations following dimensionality reduction. HVGs were selected for trajectory construction. Genes dynamically expressed along pseudotime were identified using differentialGeneTest(), which fits a generalized additive model (GAM) to model gene expression as a smooth function of pseudotime; genes with *p* < 0.01 were considered trajectory‐associated. These genes were clustered and visualized in a heatmap to pinpoint stage‐specific molecular modules.

### 2.15 Cell–Cell Communication Network Inference

Cell–cell communication analysis was carried out via the R package ‘CellChat’ (v 2.2.0) [35]. All annotated cell types were included to infer ligand‐receptor interactions. The built‐in human ligand‐receptor database (CellChatDB.human) was loaded for reference. The identify Over Expressed Genes function, identify Over Expressed Interactions, compute Commun Prob, and compute Commun Prob Pathway were employed. Interaction probabilities were estimated using a mass‐action model integrating ligand, receptor, and co‐factor expression, with significance assessed by permutation testing (1000 permutations; *p* < 0.05). Global communication strength across cell types was visualized via circle plots generated by net Visual_circle. To dissect the signalling roles of key cell type, bubble plots were generated to display incoming signals and outgoing signals. Additionally, the signalling activity of all cell types was quantified by their outgoing and incoming communication strengths. Results were visualized in a scatter.

### 2.16 Transcription Factor Activity Analysis

Regulon activity analysis was executed via the R package ‘dorothea’ (v 1.14.1) and the DoRothEA database [36]. Only high‐confidence (confidence levels A/B/C) TF–target gene interactions were retained. TF activity scores for individual cells were inferred using the VIPER algorithm, reflecting the regulatory strength of each TF. For downstream analysis, the top 200 TFs exhibiting the largest changes in mean activity (with standard deviations) across experimental groups were selected. Additionally, the AUCell algorithm was applied to evaluate the enrichment of TF target gene sets in individual cells, quantifying regulon activity. Cell‐type specificity of regulons was quantified using Regulon Specificity Score (RSS), based on Jensen–Shannon divergence, measuring the degree to which regulon activity is concentrated in specific cell types. The top 5 TFs with the highest RSS in the macrophage cluster were selected for downstream analysis.

### 2.17 RT‐qPCR

To authenticate the expression levels of key genes identified through bioinformatic analyses, RT‐qPCR was performed on clinical samples. Biological samples were collected as PBMCs from 65 pSS patients and 30 healthy controls at the Second Hospital of Shanxi Medical University. All pSS patients met the 2016 American College of Rheumatology/European League Against Rheumatism (ACR/EULAR) classification criteria for pSS. Healthy controls had no history of autoimmune diseases or infectious diseases, were negative for autoantibodies (ANA, anti‐Ro/SSA, anti‐La/SSB), and had no clinical signs of sicca. All participants gave written informed consent. The demographic and clinical characteristics of the study cohort are presented in Table 1. The research received ethical approval from the Ethics Committee of the Second Hospital of Shanxi Medical University (ethics approval number: (2023) YX No. 068; approval date: March 7, 2023). Total RNA was extracted from the samples using TRIzol reagent (Vazyme, R401‐01, China) strictly following the manufacturer's protocol. Researchers measured the concentration and purity of the isolated RNA via a NanoPhotometer N50 (Implen, Germany). Reverse transcription was carried out to synthesize first‐strand cDNA using the HifairIII 1st Strand cDNA Synthesis SuperMix Kit (Yeasen Biotechnology, Catalogue No. 11141ES60, China), according to the manufacturer's instructions. The resulting cDNA products were diluted 5–20 times with DNase/RNase‐free water prior to qPCR. qPCR reactions were performed using the 2 × Universal Blue SYBR Green Master Mix (Servicebio, G3326‐05, China) on a CFX Connect Real‐Time PCR Detection System (BIO‐RAD, XLFZ006, USA). The specific primer sequences used for each target gene were detailed in Supplementary Table 2. Amplification specificity for each reaction was confirmed by analysing the melting curve. All reactions were performed in technical triplicates to ensure reproducibility. The relative expression levels of genes were calculated via the 2^−ΔΔCt^ method, with GAPDH serving as the internal reference gene. Centrifugation steps during RNA extraction and cDNA preparation were performed using an H1 16KR high‐speed centrifuge (Xiangyi Instrument, XLSY010, China) and an SMC5000F mini centrifuge (SCILOGEX, USA). Statistical analysis of the RT‐qPCR data was conducted using GraphPad Prism software (v 10.0) [37].

**TABLE 1:** Clinical characteristics of patients with pSS and healthy controls.

Characteristics	Patients with pSS (*n* = 65)	Healthy controls (*n* = 30)
Female, *n* (%)	54 (83.1%)	23 (76.7%)
Age (years), mean ± SD	52.3 ± 10.1	50.8 ± 9.5
Disease duration (months), mean ± SD	46.15 ± 30.19	—
Anti‐Ro/SSA antibodies, *n* (%)	37 (56.9%)	—
Anti‐La/SSB antibodies, *n* (%)	22 (33.8%)	—
Dry mouth, *n* (%)	60 (92.3%)	—
Dry eye, *n* (%)	54 (83.1%)	—
Arthritis, *n* (%)	15 (23.1%)	—

Abbreviations: pSS, primary Sjögren's syndrome; SD, standard deviation; SSA, Sjögren's syndrome‐related antigen A; SSB, Sjögren's syndrome‐related antigen B.

### 2.18 Statistical Analysis

Bioinformatics analyses were executed via R software (v 4.2.2). For in silico analyses, two‐group comparisons of gene expression and immune scores were performed using the Wilcoxon rank‐sum test to satisfy statistical assumptions. Differences in gene expression levels between the pSS samples and controls in the RT‐qPCR were assessed using the *t*‐test (*p* < 0.05).

## 3 Results

### 3.1 Identification of IFNG‐Associated Genes and Functional Enrichment in pSS

**FIGURE 1:**
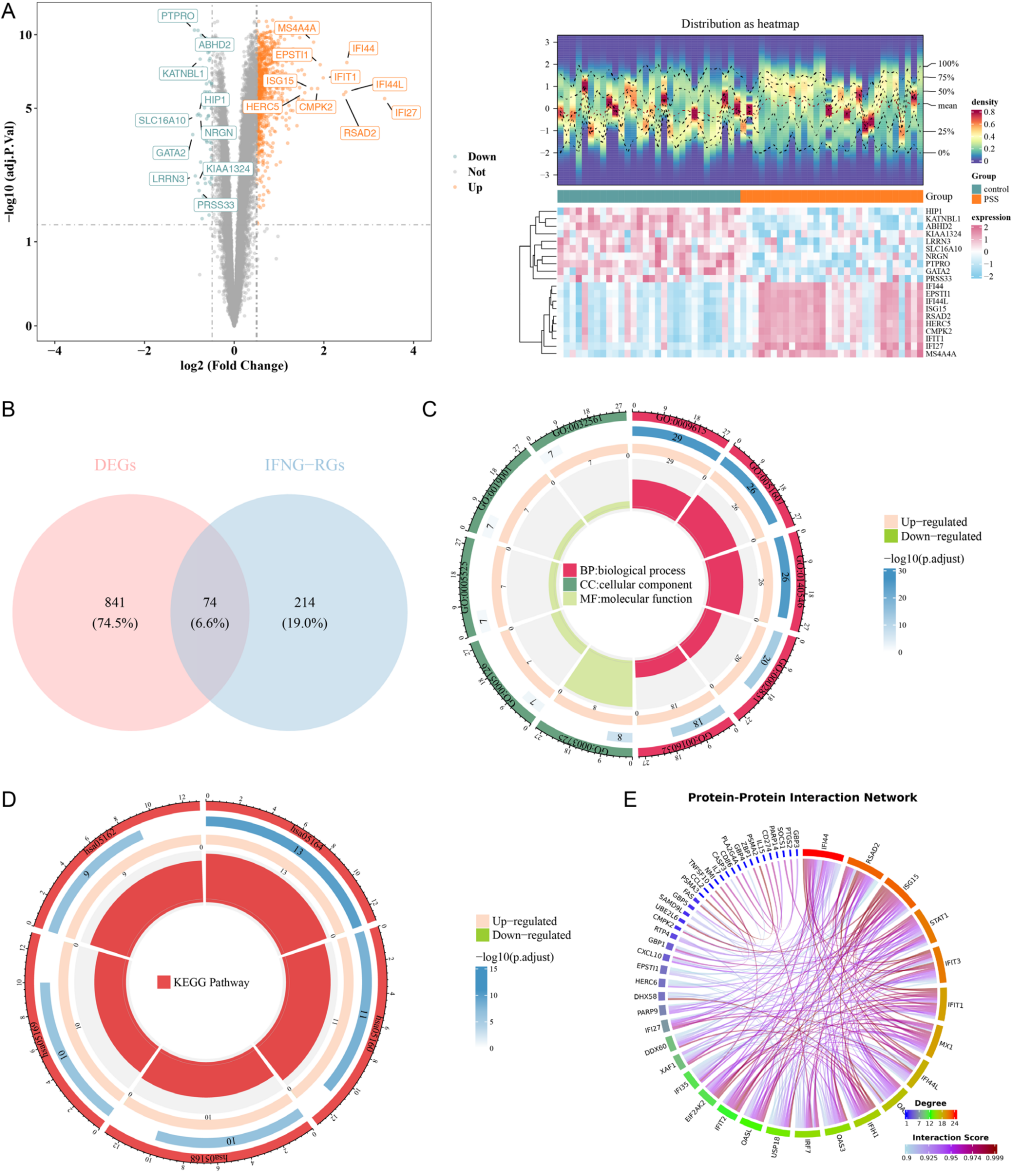
Identification of IFNG‐associated genes and functional enrichment in pSS. (A) Differential expression analysis of transcriptomic data, showing a volcano plot of differentially expressed genes (DEGs) between comparison groups. Significantly upregulated and downregulated genes are highlighted. The accompanying heatmap displays the expression patterns of representative DEGs across samples, illustrating clear transcriptional differences between groups. (B) Venn diagram illustrating the overlap between DEGs and IFNG‐related genes (IFNG‐RGs), identifying a subset of candidate genes potentially associated with IFNG signalling in primary Sjögren's syndrome (pSS). (C) Gene Ontology (GO) enrichment analysis of the identified candidate genes, summarizing the most significantly enriched biological processes, cellular components, and molecular functions potentially involved in disease‐related mechanisms. (D) Kyoto Encyclopedia of Genes and Genomes (KEGG) pathway enrichment analysis of candidate genes, highlighting key signalling pathways potentially associated with IFNG‐related molecular mechanisms in pSS. (E) Protein–protein interaction (PPI) network constructed based on candidate genes, revealing potential functional interactions and identifying core network modules that may play central roles in IFNG‐associated biological processes.

Subsequently, differential gene expression analysis identified 915 DEGs, comprising 857 upregulated and 58 downregulated genes in pSS patients (adjusted *p* < 0.05, |log2FC| > 0.5) (Figure 1A). To refine genes functionally linked to IFNG‐driven pathways, DEGs were intersected with IFNG‐RGs, yielding 74 candidate genes (Figure 1B). GO analysis revealed that these genes were significantly enriched in 195 BP terms, such as response to virus, defence response to virus, and regulation of response to biotic stimulus, as well as 17 MF terms such as double‐stranded RNA binding and cytokine receptor binding (Figure 1C, Supplementary Table 3). KEGG pathway analysis further associated these genes with 19 pathways, with the most significant being viral infection pathways (Hepatitis C, etc.) and the NOD‐like receptor signalling pathway (Figure 1D, Supplementary Table 4). PPI analysis revealed tight candidate gene connectivity. With confidence ≥ 0.9, the network contained 49 nodes and 187 edges (Figure 1E). These results collectively indicated that IFNG dysregulation in pSS might drive a pronounced antiviral and inflammatory transcriptional program. This revealed IFNG as a potential orchestrator in pSS pathogenesis.

### 3.2 Identification of Key Genes

**FIGURE 2:**
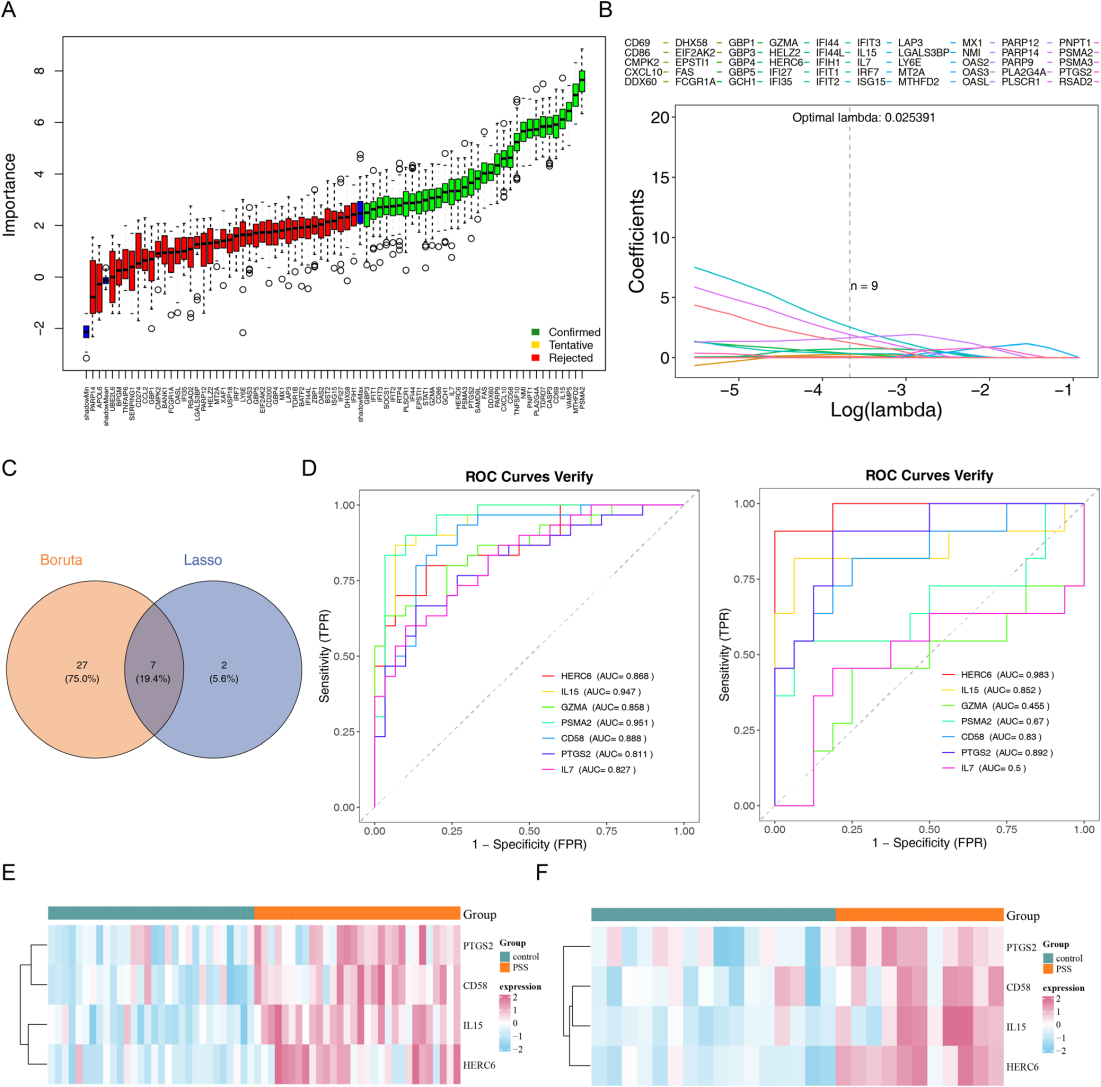
Machine learning‐based identification of key genes. (A) Boruta feature selection analysis based on random forest algorithm, used to identify all‐relevant variables associated with the outcome. Important and tentative features were retained after iterative comparison with shadow features, ensuring robust feature screening. (B) LASSO (least absolute shrinkage and selection operator) regression analysis showing coefficient profiles of candidate variables under different penalty strengths. The optimal lambda value was determined via cross‐validation, and features with non‐zero coefficients were selected for model construction. (C) Intersection of genes identified by Boruta and LASSO analyses, highlighting the overlapping genes considered as robust key candidate genes for subsequent analysis. (D) Receiver operating characteristic (ROC) curve analysis evaluating the diagnostic performance of the selected feature genes in distinguishing disease and control samples, demonstrating their predictive accuracy. (E, F) Independent validation of feature gene expression in the training cohort (E) and validation cohort (F), confirming the consistency and robustness of expression patterns across datasets.

To confirm robust key genes for pSS, two ML algorithms were implemented. Initially, the Boruta algorithm was employed to evaluate the importance of all candidate genes. This iterative process eliminated non‐essential features by comparing them with randomized shadow attributes, resulting in the selection of 34 feature genes with high relevance to pSS pathogenesis (Figure 2A). Subsequently, LASSO regression analysis was performed using 5‐fold cross‐validation to ensure stability, the optimal lambda value (lambda min = 0.025391) was determined based on minimal cross‐validated error. This step identified 9 genes, including HECT and RLD Domain‐Containing E3 Ubiquitin Protein Ligase Family Member 6 (HERC6), Interleukin15 (IL15), Granzyme A (GZMA), Z‐DNA Binding Protein 1 (ZBP1), Proteasome Subunit Alpha 2 (PSMA2), Bisphosphoglycerate Mutase (BPGM), Cluster of Differentiation 58 (CD58), prostaglandin‐endoperoxide synthase 2 (PTGS2), Interleukin 7(IL7), with non‐zero coefficients (Figure 2B). Integration of results from both methods yielded 7 overlapping genes as key genes, including HERC6, IL15, GZMA, PSMA2, CD58, PTGS2 and IL7 (Figure 2C). The discriminatory power of these genes was rigorously validated using ROC analysis. In the training cohort, all 7 genes exhibited AUC values > 0.7. In the validation cohort, HERC6, IL15, CD58 and PTGS2 maintained AUC values > 0.7, confirming their robust diagnostic capacity (Figure 2D). In addition, HERC6, IL15, CD58, and PTGS2 were consistently upregulated in pSS patients compared to healthy controls across both training and validation cohorts (Figure 2E,F). Their significant overexpression and sustained discriminatory power across two cohorts highlight their potential utility in clinical diagnostics and their biological relevance to pSS mechanisms.

### 3.3 Development and Validation of an IFN‐Gamma Response Gene Signature‐Based Diagnostic Model

**FIGURE 3:**
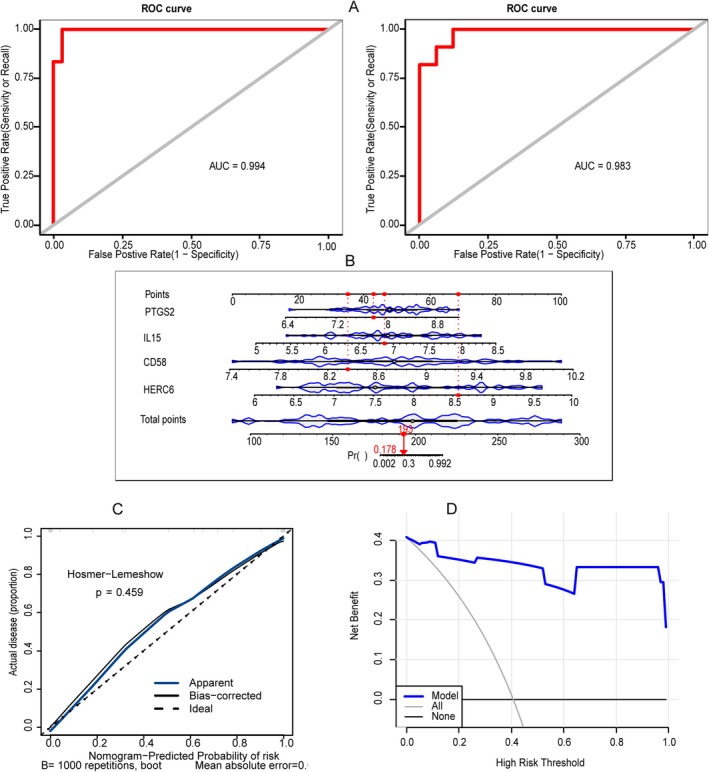
Diagnostic model performance. (A) Receiver operating characteristic (ROC) curve analysis of the IFNG‐RGS score, assessing its discriminatory ability to distinguish primary Sjögren's syndrome (pSS) patients from controls. The area under the curve (AUC) indicates the overall diagnostic accuracy of the model. (B) Nomogram constructed based on the IFNG‐RGS score for individualized risk prediction of pSS, providing a quantitative tool for estimating disease probability at the individual level. (C) Calibration curve of the nomogram, evaluating the agreement between predicted probabilities and observed outcomes. A closer fit to the diagonal line indicates better model calibration performance. (D) Decision curve analysis (DCA) assessing the clinical utility of the predictive model across a range of threshold probabilities, demonstrating the net benefit of the IFNG‐RGS‐based model compared with default strategies.

To establish a clinically applicable diagnostic tool for pSS, an IFNG‐RGS score was computed for each sample grounded in expression levels of key genes. The diagnostic performance of this score was determined via ROC analysis. In the training cohort, the model achieved an AUC of 0.994, demonstrating exceptional discriminative capacity to distinguish pSS patients from healthy controls. Robust generalizability was confirmed in an independent validation cohort, where the model maintained an AUC > 0.90 (Figure 3A). To enhance clinical interpretability, a nomogram was constructed by integrating the expression levels of the key genes. This visual tool assigned individual points for each gene's expression level, with the cumulative “total points” directly correlating with the predicted risk of pSS. For instance, a representative sample with total points of 193 corresponded to a 17.8% probability of pSS diagnosis (Figure 3B). The calibration curve showed close alignment between predicted probabilities and actual observed outcomes. This was statistically supported by a not meaningful Hosmer–Lemeshow test result (*p* = 0.459) (Figure 3C). DCA further quantified the clinical utility of the nomogram. The model demonstrated substantially higher net benefit across a wide range of threshold probabilities compared to ‘treat‐all’ or ‘treat‐none’ strategies, supporting its potential value for clinical decision‐making (Figure 3D). The IFNG‐RGS score and nomogram represent a powerful and clinically applicable diagnostic tool for pSS, and provided a practical framework for translating complex gene expression signatures into actionable clinical assessments for pSS diagnosis.

### 3.4 Immune Microenvironment Characterization and Pathway Alterations in pSS

**FIGURE 4:**
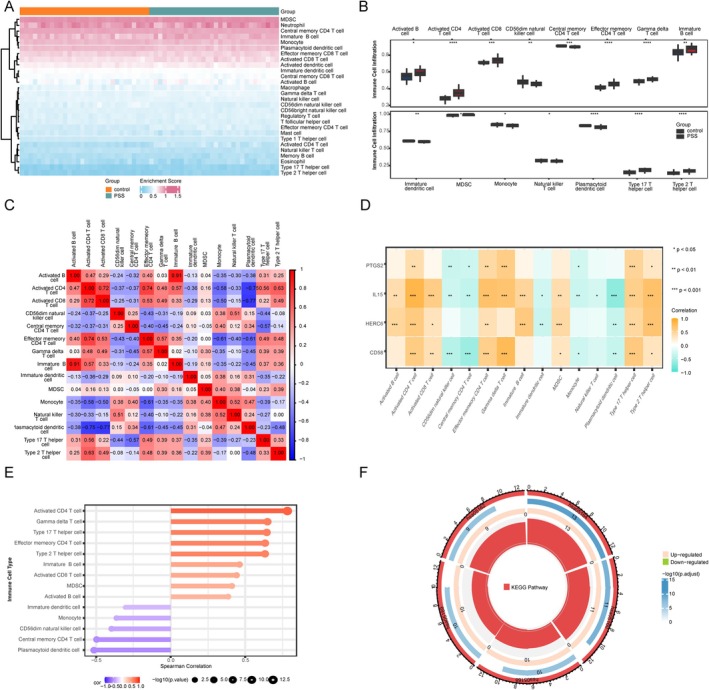
Immune microenvironment dysregulation. (A) Immune cell infiltration landscape across samples, illustrating the overall composition and abundance of different immune cell populations within the immune microenvironment. (B) Differential analysis of immune cell infiltration between groups, identifying significantly altered immune cell subsets associated with disease status or IFNG‐RGS score stratification. *p < 0.05, **p < 0.01, ***p < 0.001, ****p < 0.0001. (C) Correlation analysis among infiltrating immune cell types, revealing co‐infiltration patterns and potential regulatory relationships within the immune microenvironment. (D) Correlation analysis between key genes and immune cell infiltration levels, suggesting potential associations between candidate genes and specific immune cell populations. *p < 0.05, **p < 0.01, ***p < 0.001. (E) Correlation analysis between IFNG‐RGS score and immune cell infiltration, demonstrating the relationship between the risk signature and immune microenvironment remodelling. (F) KEGG pathway enrichment analysis based on immune‐related alterations, highlighting key signalling pathways potentially involved in immune microenvironment dysregulation in pSS.

To delineate the immune landscape of pSS, xCell‐derived enrichment scores of 28 immune cell types were computationally estimated from peripheral blood transcriptomic data. Fibroblasts, CD4 memory T cells, and macrophages exhibited higher infiltration levels (Figure 4A). Comparative analysis revealed 36 immune cell types with significantly altered infiltration between pSS patients and healthy controls (*p* < 0.05). Specifically, common lymphoid progenitors (CLPs), activated dendritic cells (aDCs), macrophages, macrophages M1, and Th2 cells were markedly elevated in pSS patients. Conversely, astrocytes, CD4+ naive T cells, epithelial cells, and pericytes were significantly reduced (Figure 4B). Correlation analysis further uncovered coordinated immune cell dynamics. B cells strongly correlated with naive B cells (cor = 0.99, *p* < 0.05), class switched memory B cells (cor = 0.92, *p* < 0.05), and pro‐B cells (cor = 0.91, *p* < 0.05). CD4+ memory T cells were positively associated with smooth muscle cells (cor = 0.89, *p* < 0.05). Astrocytes inversely correlated with CLPs (cor = −0.72, *p* < 0.05) and activated CD8 T cells (cor = −0.77, *p* < 0.05). Macrophages negatively associated with CD4+ naive T cells (cor = −0.69, *p* < 0.05) (Figure 4C, Supplementary Table 5). All four genes positively correlated with CLPs, macrophages, and melanocytes (cor > 0.3, *p* < 0.01). These genes were inversely associated with astrocytes and CD4+ naive T cells (cor < −0.3, *p* < 0.01). PTGS2, IL15, and CD58 negatively related to mesangial cells (cor < −0.3, *p* < 0.01) (Figure 4D, Supplementary Table 6). In addition, the IFNG‐RGS score exhibited positive correlations with macrophages, Th2 cells, CD4+ memory T cells, plasma cells, melanocytes, CLPs, and aDCs (cor > 0.4, *p* < 0.0001). Negative correlations were detected between the IFNG‐RGS score and pericytes, astrocytes (cor < −0.4, *p* < 0.0001) (Figure 4E). Furthermore, 106 significantly altered pathways were identified. Some pathways were upregulated in pSS patients, such as oxidative phosphorylation, SNARE interactions in vesicular transport, and basal TFs. Downregulated pathways encompassed arachidonic acid metabolism, fructose and mannose metabolism, and ERBB signalling (Figure 4F, Supplementary Table 7). These findings collectively demonstrated a profoundly dysregulated immune microenvironment in pSS. The strong correlations between the key interferon‐response genes and the IFNG‐RGS score with specific immune cell types revealed the potential role of IFN‐γ signalling in driving this immune imbalance. Furthermore, the widespread metabolic and signalling pathway alterations suggested broader cellular reprogramming contributed to pSS pathogenesis beyond overt immune activation.

### 3.5 Functional Characterization and Regulatory Networks of Key Genes

**FIGURE 5:**
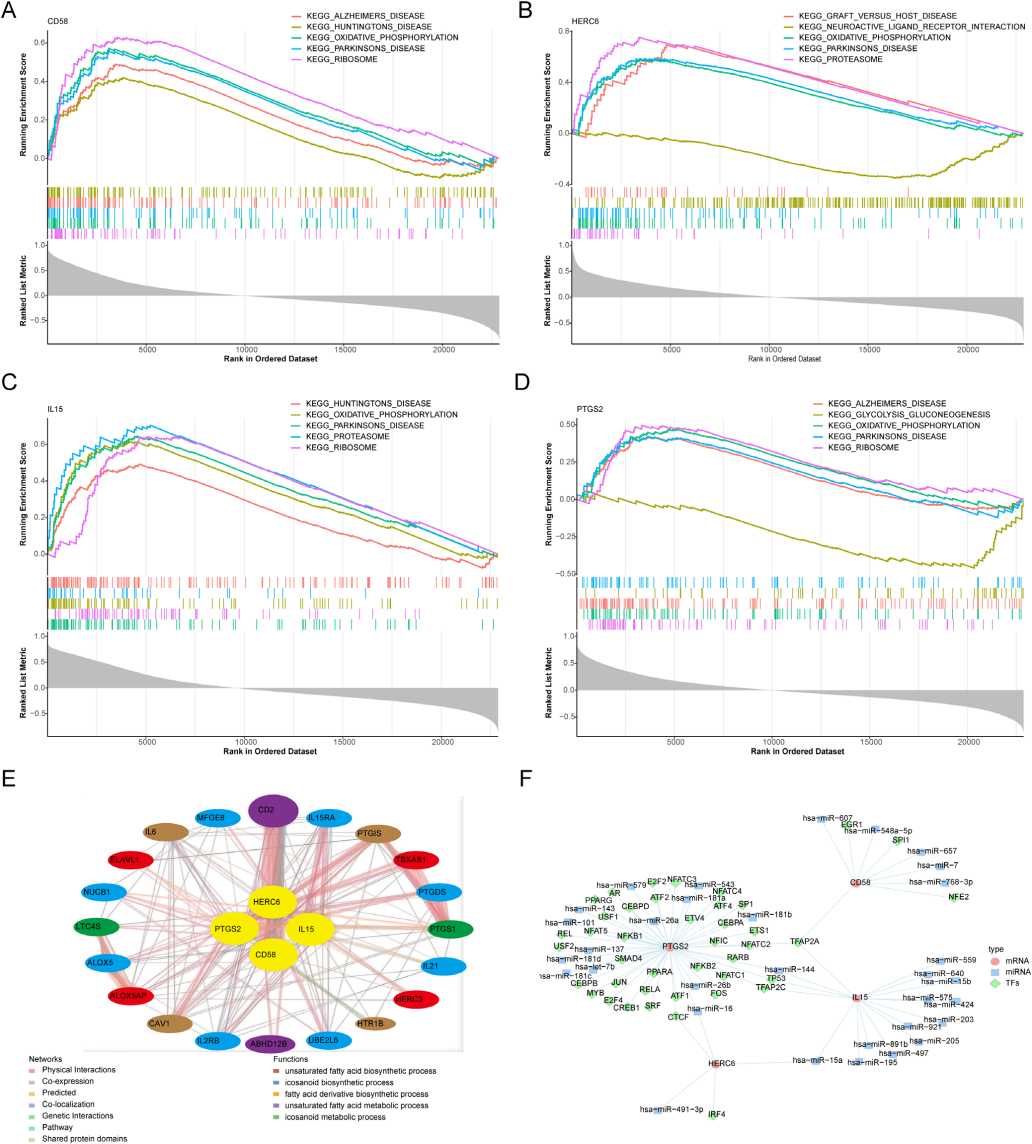
Functional and regulatory networks. (A–D) Gene set enrichment analysis (GSEA) of key genes including CD58 (A), HERC6 (B), IL15 (C), and PTGS2 (D), respectively. CD58‐associated pathways are mainly enriched in immune cell interaction and immune regulation processes. HERC6 shows enrichment in antiviral and interferon‐related signalling pathways. IL15 is primarily associated with immune activation and lymphocyte regulation pathways. PTGS2 is enriched in inflammation‐related signalling pathways, suggesting its involvement in immune‐mediated inflammatory responses in primary Sjögren's syndrome (pSS). (E) GeneMANIA‐based functional interaction network illustrating gene–gene relationships, including co‐expression, physical interactions, shared protein domains, and pathway associations among key genes. (F) Transcription factor (TF)–miRNA regulatory network constructed to explore upstream regulatory mechanisms of key genes, highlighting potential transcriptional and post‐transcriptional regulators in pSS.

Functional enrichment analysis revealed distinct pathway associations for each key gene. CD58 was significantly enriched in 11 pathways, with prominent involvement in ribosome, oxidative phosphorylation and adherens junction (Figure 5A, Supplementary Table 8). HERC6 demonstrated broader functional connectivity, showing enrichment across 64 pathways including neuroactive ligand‐receptor interaction and proteasome (Figure 5B, Supplementary Table 9). Similarly, IL15 participated in 69 pathways, notably oxidative phosphorylation and ribosome (Figure 5C, Supplementary Table 10). PTGS2 exhibited more focused associations with 10 pathways, primarily glycolysis, gluconeogenesis and oxidative phosphorylation (Figure 5D, Supplementary Table 11). GeneMANIA network analysis further elucidated functional cooperativity among the key genes. Key genes and their related genes formed interconnected modules through physical interactions, co‐expression, and co‐localization. These modules were functionally annotated to lipid metabolism pathways, particularly unsaturated fatty acid biosynthetic process, and icosanoid metabolic process, indicating coordinated regulation of immunometabolic functions (Figure 5E). Furthermore, transcriptional and post‐transcriptional regulatory analysis identified potential master regulators. hsa‐miR‐15a was predicted to simultaneously target both HERC6 and IL15, suggesting miRNA‐mediated co‐regulation of interferon response genes. The TFAP2A emerged as a putative regulator of IL15, PTGS2 and CD58, implying hierarchical control of multiple key genes through a common transcriptional hub (Figure 5F). These findings delineated both distinct and shared functional roles for the key genes. The network analyses revealed their potential functional cooperativity within lipid metabolism pathways and uncovered potential upstream regulatory mechanisms, including miRNA‐mediated co‐regulation and hierarchical transcriptional control, providing insights into the coordinated dysregulation underlying pSS immunopathology.

### 3.6 Single‐Cell Transcriptomic Profiling Revealed Macrophage‐Centric Dysregulation in pSS

**FIGURE 6:**
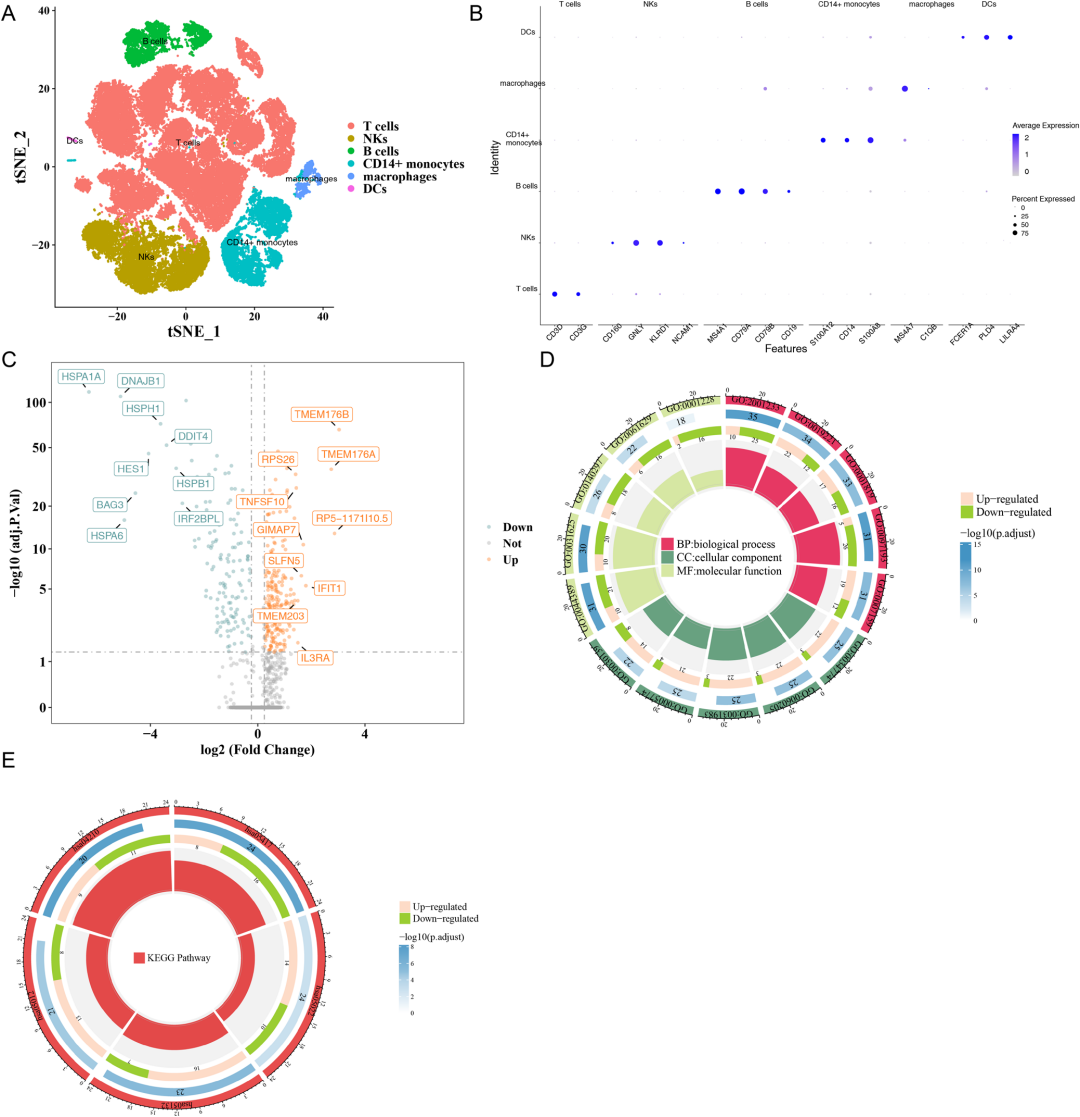
Single‐cell transcriptomic profiling. (A) Cell type annotation of identified clusters based on canonical marker genes, enabling classification of major immune and stromal cell populations. (B) Expression distribution of highly variable genes (HVGs), highlighting genes with significant cell‐to‐cell variability that contribute to cellular heterogeneity. (C) Identification of differentially expressed genes (DEGs) in macrophages, revealing transcriptional alterations specific to this cell population. (D, E) Functional enrichment analysis of macrophage DEGs, including Gene Ontology (GO) and Kyoto Encyclopedia of Genes and Genomes (KEGG) pathway analyses, indicating that these genes are enriched in immune‐related and inflammation‐associated biological processes and signalling pathways.

Analysis of scRNA‐seq data was performed to delineate the cellular heterogeneity in pSS. After rigorous quality control and normalization, 2000 HVGs were selected for dimensionality reduction. PCA demonstrated consistent cellular distribution across samples, with the top 20 PCs selected for subsequent clustering (Supplementary Figure 1). Unsupervised clustering at a resolution of 0.3 identified 17 distinct clusters (Supplementary Figure 2A). Cross‐referencing with established marker genes from literature and the CellMarker database enabled annotation of six major immune cell types, including T cells, NKs, B cells, and so forth (Figure 6A). Quantification of cellular proportions revealed T cells as the predominant population, followed by NKs, across both pSS patients and healthy controls. Cell type‐specific expression patterns were observed for highly variable genes (Figure 6B). Evaluation of key gene expression demonstrated that CD58 was broadly expressed across multiple cell types, whereas HERC6 exhibited restricted expression. Critically, macrophages showed the highest average expression levels of all four key genes, identifying macrophages as pivotal in pSS pathogenesis (Supplementary Figure 2B). Differential gene expression analysis of macrophages identified 386 dysregulated genes in pSS patients (241 upregulated, 145 downregulated) (Figure 6C). Functional enrichment analysis disclosed these genes were markedly linked to some BP terms, such as regulation of apoptotic signalling, cytokine‐mediated signalling pathways, and leukocyte cell–cell adhesion; CC terms, such as secretory granule lumen, vesicular compartments, and endocytic vesicles; and MF terms, such as ubiquitin‐protein ligase binding and transcription factor activity (Figure 6D, Supplementary Table 8). KEGG pathway analysis further implicated these genes in lipid and atherosclerosis, neurodegenerative pathways, and apoptosis (Figure 6E, Supplementary Table 9). These findings positioned macrophages as potential central orchestrators in pSS pathogenesis.

### 3.7 Trajectory Analysis Revealed Developmental Dynamics and Cell–Cell Communication Networks in pSS

**FIGURE 7:**
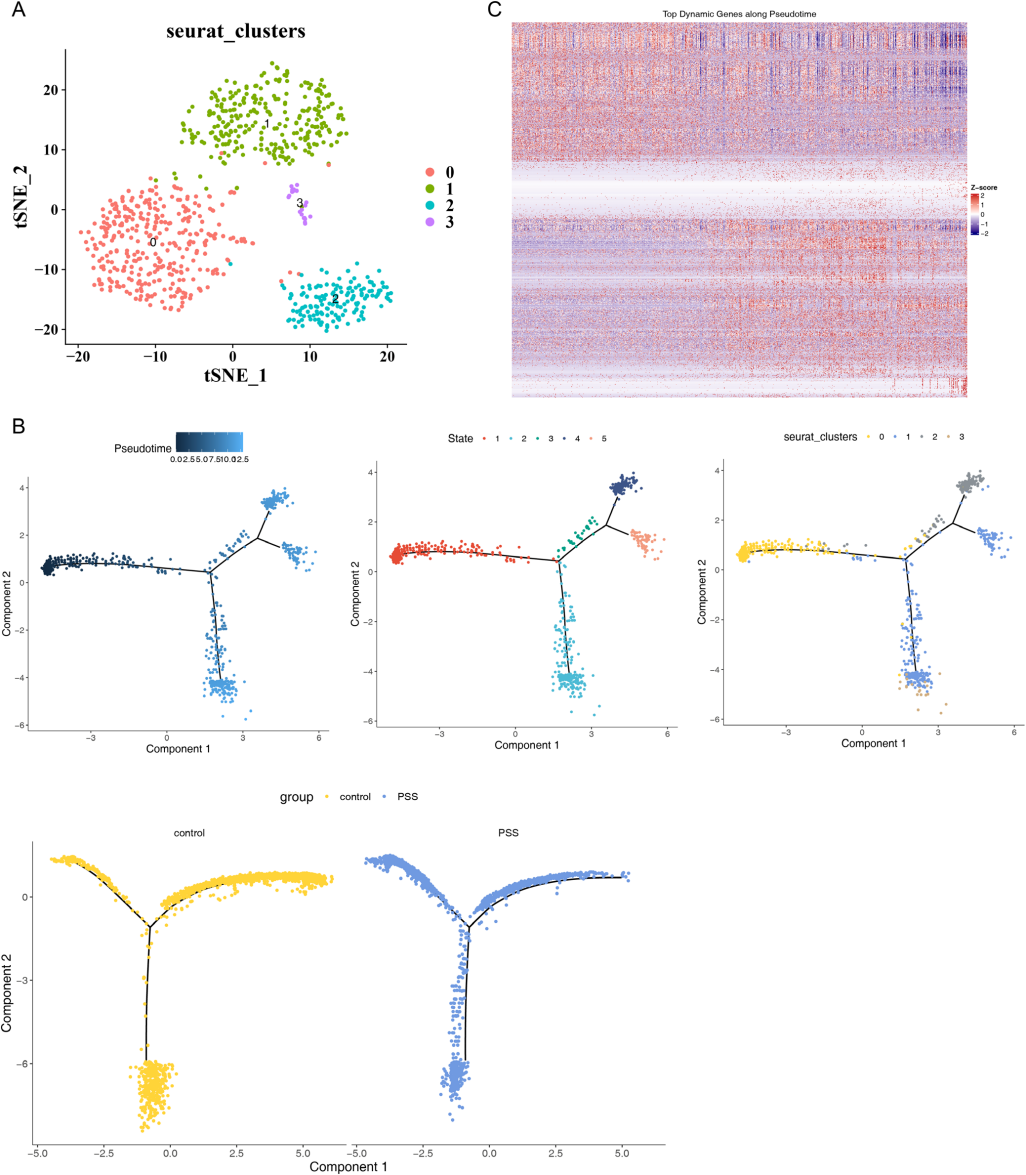
Macrophage differentiation trajectory analysis. (A) Identification of macrophage subclusters based on single‐cell transcriptomic profiling, revealing distinct macrophage subpopulations with heterogeneous transcriptional states. (B) Pseudotime trajectory analysis of macrophage differentiation, illustrating the dynamic progression and potential developmental continuum among macrophage subclusters along inferred differentiation trajectories. (C) Stage‐specific gene expression dynamics along pseudotime, showing the temporal changes in key gene expression patterns during macrophage state transitions and highlighting genes associated with different differentiation stages.

**FIGURE 8:**
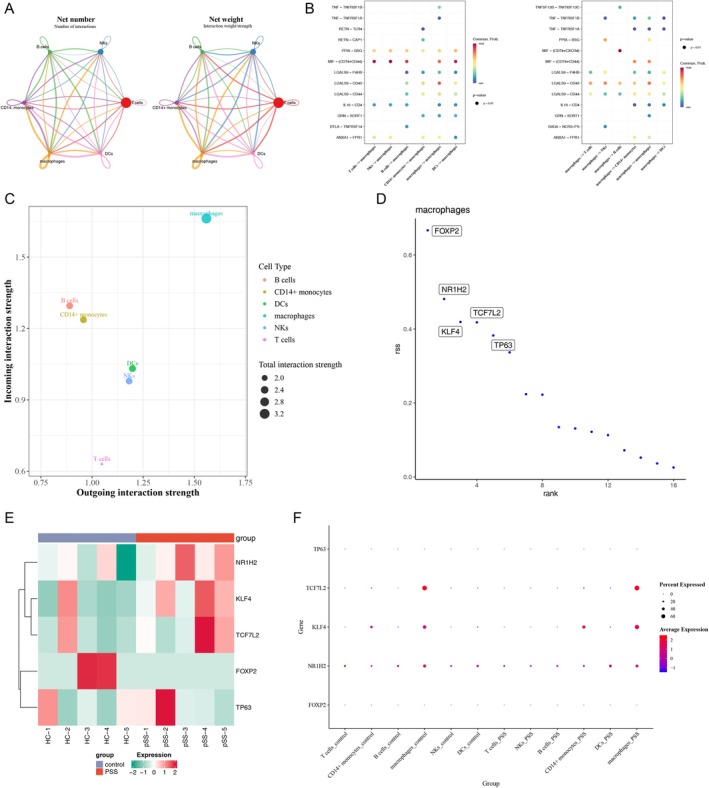
Macrophage communication and regulatory network. (A) Global cell–cell interaction network among immune cell populations. (B) Macrophage‐centred ligand–receptor signalling network. (C) Relative signalling pathway activity across macrophage states. (D) Ranking plot showing prioritized genes based on feature importance scores in macrophage‐related analysis. (E) Heatmap displaying differential expression of selected genes across control and disease groups. Rows represent genes and columns represent samples. (F) Dot plot illustrating gene expression levels and expression proportions across different cell populations or conditions. Dot size indicates percentage of expressing cells, and colour intensity represents average expression.

To delineate the state transition of macrophages during pSS progression, macrophages were reclustered into four distinct subpopulations (Figure 7A). Pseudotime trajectory analysis demonstrated a progressive differentiation path from early‐stage to mature states. The trajectory was partitioned into five states: state 1 represented the initial undifferentiated state, state 3 an intermediate transitional phase, and states 2/4/5 denoted terminal differentiation. Subpopulation mapping revealed that cluster 0 marked the developmental origin, cluster 1 aligned with late‐stage differentiation (state 5 and state 2‐late), while cluster 3 corresponded to terminal maturation in state 2. Critically, pSS patient samples exhibited significant enrichment in early differentiation stages compared to controls, suggesting developmental arrest or accelerated entry into differentiation pathways (Figure 7B). Transcriptomic dynamics along the trajectory identified 983 stage‐specific genes dynamically activated during macrophage maturation (Figure 7C). Cell–cell communication analysis revealed extensive ligand‐receptor interactions across immune cell types. Macrophages and CD14^+^ monocytes demonstrated the strongest interactions, both in interaction frequency and communication weight (Figure 8A). As signal receivers, macrophages primarily communicated with T cells, B cells, and dendritic cells through the MIF−(CD74 + CD44) axis. As signal senders, they engaged B cells via MIF−(CD74 + CD44) and interacted with NK cells/macrophages through LGALS9 − CD45 (Figure 8B). Macrophages exhibited the highest overall signalling activity (outgoing/incoming strength), whereas T cells showed minimal communication capacity (Figure 8C). Regulon specificity analysis identified TCF7L2, KLF4, and NR1H2 as top‐ranked TFs with macrophage‐biased activity in pSS (Figure 8D). These TFs were significantly upregulated in pSS macrophages compared to controls (Figure 8E), with TCF7L2 and KLF4 demonstrating particularly enriched expression within macrophages (Figure 8F). These findings collectively indicate a dysregulated macrophage differentiation trajectory in pSS, characterized by an accumulation of cells in early developmental stages. Concurrently, macrophages emerged as central communication hubs within the immune microenvironment, engaging in robust bidirectional signalling, particularly via MIF and LGALS9 pathways, and exhibit heightened activity of specific TFs that likely drive their pathogenic state and functional interactions in the disease. This might position macrophages as key orchestrators of immune dysregulation through both altered differentiation dynamics and enhanced intercellular communication in pSS.

### 3.8 Validation of Key Gene Expression via RT‐qPCR

**FIGURE 9:**
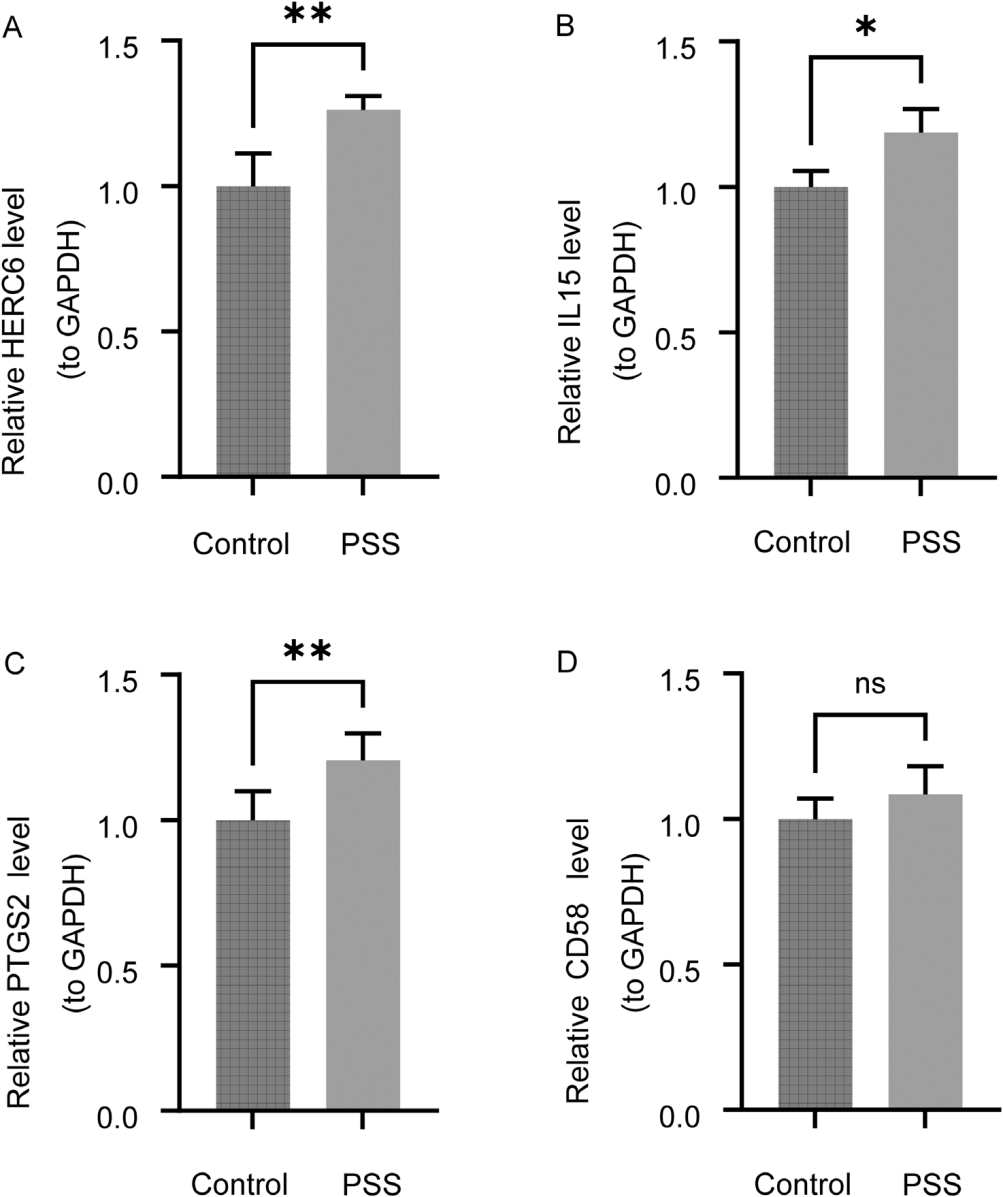
Validation of key gene expression levels in pSS patients and healthy controls using RT‐qPCR. (A–C) HERC6 (A), IL15 (B), and PTGS2 (C) expression levels were significantly higher in pSS patients than in healthy controls (*p* < 0.05). (D) CD58 expression exhibited an upward trend in pSS patients but did not reach statistical significance (*p* ≥ 0.05). Statistical significance was determined by the unpaired *t*‐test (*p* < 0.05). *p*‐values: **p < 0.01, *p < 0.05; ns, not significant.

To validate the predictive findings from prior bioinformatics analyses, RT‐qPCR was carried out to quantify mRNA expression levels of HERC6, IL15, CD58, and PTGS2 in pSS patients and health controls. Significant upregulation of HERC6, IL15, and PTGS2 mRNA levels was observed in pSS patients compared to controls (*p* < 0.05) (Figure 9A–C). In contrast, CD58 expression exhibited an upward trend in pSS patients, but this difference did not reach statistical significance (Figure 9D). Collectively, these results confirmed the dysregulation of HERC6, IL15 and PTGS2 in pSS pathogenesis, aligning with initial bioinformatics predictions. The non‐significant trend in CD58 expression might suggest its potential role in disease mechanisms warranting further investigation.

## 4 Discussion

pSS is a chronic autoimmune disease characterized by exocrine gland dysfunction and systemic inflammation [2, 38]. Dysregulation of the IFN‐γ signalling pathway is considered a key factor in its pathophysiology; however, the underlying molecular and cellular mechanisms remain unclear [13, 16]. In this study, four core IFN‐γ‐related diagnostic genes—HERC6, IL15, CD58, and PTGS2—were identified, all of which were consistently upregulated in pSS patients. Subsequent functional analysis revealed their strong association with hallmark pathological features of pSS, including aberrant IFN signalling pathway activation and dysregulated immune‐inflammatory responses. Furthermore, single‐cell transcriptomics and others untangled the central role of macrophages in coordinating the disease microenvironment, providing new insights into the pathophysiology of pSS.

HERC6, an interferon‐induced E3 ubiquitin ligase, is functionally linked to the type I interferon (IFN‐I) response [39]. Studies have shown that HERC6 mediates ISGylation of STING protein, thereby promoting its stability and enhancing downstream signalling [40]. Conversely, deficiency of HERC6 leads to sustained activation of the STING‐TBK1 signalling pathway and excessive expression of interferon‐stimulated genes (ISGs) [41]. Notably, HERC6 was also found to be upregulated in PBMCs of systemic lupus erythematosus (SLE) patients, where it promotes cell death and inflammatory responses via the JAK/STAT pathway [42]. The RT‐qPCR results in this study show significant upregulation of HERC6 in pSS patients, which suggests that it may contribute to the characteristic IFN‐gamma signalling hyperactivation in pSS through similar mechanisms—by regulating STING pathway activity and ISG expression—thereby driving inflammation and tissue injury.

Interleukin‐15 (IL‐15), encoded by the IL15 gene, is a pivotal pro‐inflammatory cytokine that plays an essential role in the activation, proliferation, and function of CD8^+^ T cells and NK cells [43]. This study was validated by RT‐qPCR experiments, which revealed that IL‐15 was significantly upregulated in patients with pSS, which is consistent with previous observations of elevated IL‐15 expression in SGECs of pSS patients [44]. Both IFN‐I and IFN‐γ can induce IL‐15 expression [45]. In the context of pSS, excessive IFN‐I production is likely to act synergistically with upregulated IL‐15 to promote the expansion and activation of IFN‐γ–producing effector lymphocytes, such as CD38^+^HLA‐DR^+^ cells [43]. Furthermore, IL‐15 exerts a critical regulatory effect on IFN‐γ production by NK cells, while IFN‐γ, and in turn, potently induces IL‐15 expression in epithelial cells, forming a potential positive feedback loop that exacerbates local and systemic inflammatory responses [45, 46]. Therefore, the upregulation of IL‐15 may represent a key node linking innate immune dysregulation (IFN‐I) and adaptive immune abnormalities (T/NK cell activation and IFN‐γ production) in pSS.

CD58 (LFA‐3) is a widely expressed co‐stimulatory molecule that is essential for immune synapse formation and lymphocyte activation [47]. Previous research has shown that the interaction between CD58 and its receptor CD2 is critical for the activation of adaptive NK cells—evidenced by upregulation of CD69, CD25 and HLA‐DR—and for the production of cytokines such as IFN‐γ and TNF‐α in response to viral infection; these effects are significantly inhibited when the CD2/CD58 pathway is blocked [48]. In the autoimmune context of pSS, the upregulation of CD58 may enhance interactions between T cells or NK cells and antigen‐presenting cells—including potentially damaged glandular epithelial cells—thereby promoting the activation of effector lymphocytes and the release of pro‐inflammatory cytokines such as IFN‐γ and TNF‐α, which could exacerbate tissue inflammation and injury.

The PTGS2 gene encodes cyclooxygenase‐2 (COX‐2) [49]. Its principal catalytic product, prostaglandin E2 (PGE2), is a potent inflammatory mediator involved in various pathophysiological processes, including vasodilation, pain, fever, and the regulation of immune cell functions [50]. Upregulation of PTGS2 has been detected in patients with pSS, indicating active prostaglandin synthesis within the local inflammatory microenvironment. Infiltrating inflammatory cells serve as a major source of PGE2, and its production increases markedly in damaged tissues, such as inflamed salivary glands [49]. Activation of the PTGS2/PGE2 pathway not only directly contributes to classical signs of inflammation and tissue injury—such as pain and swelling—but may also indirectly influence the immunopathological progression of pSS by modulating the functions of immune cells, including macrophages and T cells.

Functional enrichment analysis in this study revealed that the four key genes are not only engaged in immune‐inflammatory pathways but are also closely associated with energy metabolism and cellular structural remodelling, highlighting the central role of immune‐metabolic dysregulation in pSS. CD58 was predominantly enriched in pathways related to ribosome biosynthesis, oxidative phosphorylation (OXPHOS), and adherens junctions. This finding is highly consistent with its function as a co‐stimulatory molecule—effective immune synapse formation requires substantial protein synthesis (ribosomes) and sustained energy supply (OXPHOS). Enrichment in the adherens junction pathway further supports the mechanism by which CD58 promotes immune activation by enhancing adhesion between lymphocytes and target cells [48]. Notably, the oxidative stress state observed in the salivary glands of pSS patients may be associated with increased energy demands resulting from CD58‐mediated immune cell activation [51, 52]. IL‐15 was also significantly enriched in OXPHOS and ribosome pathways. As a key cytokine essential for the survival and function of CD8+ T cells and NK cells, IL‐15 signalling requires substantial biosynthetic and energetic support. Studies have shown that IL‐15 priming can reverse metabolic constraints on IFN‐gamma production in NK cells [46], and OXPHOS may serve as a critical energy source in this process. Furthermore, the potential association between IL‐15 and the immunoproteasome subunit LMP7 suggests that IL‐15 may influence T cell differentiation (e.g., Th17) through the regulation of protein degradation, thereby contributing to adaptive immune abnormalities in pSS [53].

GeneMANIA network analysis in this study revealed that HERC6, IL15, CD58, and PTGS2 form a tightly interconnected functional module through mechanisms such as physical interactions and co‐expression. This module was significantly enriched in lipid metabolic pathways, suggesting that lipid metabolism serves as a central hub linking these four genes. Notably, lipid mediators such as PGE2, a product of PTGS2, can amplify inflammatory signals and contribute to tissue injury [54, 55]. Based on these findings, an integrated immunopathogenic model of pSS was constructed: HERC6‐mediated IFN‐1 signalling promotes IL‐15 expression and lymphocyte activation. Subsequently, CD58 enhances intercellular interactions and facilitates IFN‐γ release, which in turn induces PTGS2 expression and disrupts lipid metabolism, thereby aggravating inflammatory damage. This process is modulated by factors including hsa‐miR‐15a and is supported by metabolic processes throughout including OXPHOS, confirming the central role of the immune–metabolic axis in pSS.

This study identified key miRNAs such as hsa‐miR‐15a through regulatory network analysis, which was predicted to target both HERC6 and IL15. Previous studies have reported that dysregulated miRNA expression occurs in the peripheral blood and salivary glands of pSS patients, and some miRNAs may contribute to disease progression by modulating interferon‐related signalling, macrophage polarization, and inflammatory responses [14, 56, 57, 58]. These findings are consistent with previous reports and further support the biological plausibility of the identified miRNAs as candidate post‐transcriptional regulators in pSS.

This study successfully established and validated a diagnostic model for pSS based on an interferon‐γ response gene signature (IFNγ‐RGS) score, which demonstrated excellent clinical translation potential. To enhance clinical applicability, a visual nomogram was further developed. The IFNγ‐RGS score and the nomogram not only represent a novel molecular mechanism‐based diagnostic strategy for pSS but also serve as a bridge translating complex gene expression signatures into an actionable clinical assessment tool [59, 60, 61].

Through computational immune deconvolution of peripheral blood transcriptomic data using xCell, this study inferred a specific circulating immune cell composition pattern in pSS, characterized primarily by significant enrichment of CD4+ memory T cells and macrophages. These peripheral blood‐based estimates may indirectly reflect the broader immune dysregulation in pSS, though they do not directly represent immune infiltration in salivary gland tissues. Thirty‐six immune cell types exhibited significant differences between pSS and healthy controls: B cells, CD4+ memory T cells, and others were significantly increased, whereas astrocytes, CD4+ naive T cells, epithelial cells, and pericytes were markedly reduced. These findings are highly consistent with the core pathological features of pSS, which involve B cell hyperactivation and T cell infiltration [62, 63]. Key interferon response genes and the IFNγ‐RGS score showed significant positive correlations with specific immune cell subsets, such as common lymphoid progenitors (CLPs), macrophages, and Th2 cells, and negative correlations with astrocytes and pericytes. This aligns closely with previous studies demonstrating that activated CD8+ T cells are positively linked to IFN‐γ and elevated in pSS patients [64], thereby confirming the crucial role of interferon signalling in driving characteristic T cell priming and Th17 bias in pSS. Pathway enrichment analysis further indicated that the upregulation of the OXPHOS pathway suggests energy metabolic reprogramming in effector immune cells and inflamed tissues. Conversely, the downregulation of pathways such as arachidonic acid metabolism may reflect metabolic substrate consumption and transformation in response to supporting OXPHOS. Together, these findings delineate a complex regulatory landscape in which immune and metabolic networks are intricately intertwined in pSS.

In this study, scRNA‐seq was employed to systematically characterize the cellular heterogeneity in pSS. It was revealed that macrophages serve as the central cellular hub expressing key genes and play a pivotal role in immunopathology. Macrophages are essential for maintaining immune homeostasis, and their functional states are tightly regulated by microenvironmental signals [65]. Differential gene expression analysis demonstrated that macrophages in pSS exhibit a highly activated and dysfunctional state, with 386 DEGs enriched in pathways related to apoptosis regulation, cytokine signalling, and lipid metabolism. These findings are consistent with clinical observations: a significant increase in pro‐inflammatory M1 macrophages was detected in both peripheral blood and salivary glands of pSS patients, which positively correlated with serum IgG levels, while anti‐inflammatory M2 macrophages were reduced [66]. This confirms that an imbalance in macrophage polarization is closely associated with disease activity.

Pseudotime trajectory analysis revealed that samples from pSS patients were predominantly enriched in the early stages of the differentiation trajectory, suggesting an early blockade or abnormal acceleration in the macrophage differentiation process, leading to the accumulation of specific intermediate‐state cells. This observation aligns with previous reports of imbalanced macrophage polarization in pSS patients [66]. Existing studies indicate that bone marrow‐derived growth factors promoting M2 polarization can ameliorate the disease, while LTK deficiency‐induced M2 polarization also exerts a protective effect, further supporting the critical role of macrophage differentiation/polarization status in the pathological progression of pSS [67, 68]. Cell–chat analysis confirmed that macrophages function as signalling hubs within the microenvironment, extensively interacting with T cells, B cells, and NK cells through the MIF–(CD74 + CD44) and LGALS9–CD45 pathways, thereby coordinating immune cell infiltration and activation. Transcriptional regulatory analysis identified three key TFs—TCF7L2, KLF4 and NR1H2—that were specifically activated in pSS macrophages. These factors are implicated in the Wnt/β‐catenin signalling pathway, regulation of macrophage polarization, and lipid metabolism–inflammation crosstalk, respectively, and may collectively drive the pathological state of macrophages and their robust intercellular communication capacity [69, 70, 71].

This study identified four key IFN‐γ‐associated genes (HERC6, IL15, CD58 and PTGS2), in pSS and elucidated their molecular mechanisms in disease pathogenesis. scRNA‐seq analysis revealed that macrophages contribute to the pathophysiology of pSS by altering differentiation, enhancing intercellular signalling, and promoting IFN‐γ‐driven gene expression, thereby providing novel insights into disease mechanisms and potential therapeutic targets. However, this study has some limitations. First, all transcriptomic and single‐cell data were derived from peripheral blood, and the absence of lesional tissue samples (e.g., salivary glands) precludes establishment direct links between peripheral molecular signals and pathological injury in target organs. Second, the reliance on public databases resulted in a lack of key clinical information, which limited stratified analyses and may have introduced selection bias. Finally, the mechanistic investigations remained at the level of transcriptomic associations without functional validation; thus, the causal relationship between the proposed molecular signatures and pathological alterations requires further confirmation.

## 5 Conclusion

In summary, this study developed an IFN‐γ–centred diagnostic model for pSS and identified four core signature genes (HERC6, IL15, CD58 and PTGS2). Our findings indicate that macrophages may play a central role in immune dysregulation, potentially through shifts in differentiation states, intensified intercellular communication, and IFN‐γ–associated transcriptional programs. Collectively, these observations offer additional insight into the immunopathological mechanisms underlying pSS and highlight candidate molecular targets for therapeutic intervention.

## Author Contributions

**Jing Zhang:** writing – review and editing, supervision. **Tian‐chi Wei:** writing – original draft, methodology, software, validation, formal analysis. **Lei Shi:** methodology, data curation, validation, writing – original draft, investigation, software, project administration, formal analysis.

## Funding

The authors have nothing to report.

## Ethics Statement

This study was conducted in accordance with the principles of the Declaration of Helsinki. Ethical approval was obtained from the Ethics Committee of the Second Hospital of Shanxi Medical University (approval no. (2023) YX No. 068; Date: March 7, 2023). Written informed consent was obtained from all participants prior to sample collection.

## Consent

All participants provided written informed consent for the use of their samples and anonymized data for this research and its publication. No identifiable personal information was included in the study.

## Conflicts of Interest

The authors declare no conflicts of interest.

## Supporting information


**Supplementary Figure 1.** Quality control and dimensionality reduction of scRNA‐seq data. (A, B) Violin plots showing the distributions of nFeature_RNA, nCount_RNA, and percent.mt before and after quality control filtering, respectively. nFeature_RNA indicates the number of detected genes per cell, nCount_RNA indicates the total UMI counts per cell, and percent.mt indicates the percentage of mitochondrial gene expression. (C) Identification of the top 2000 highly variable genes for downstream dimensionality reduction analysis. Red dots represent highly variable genes, whereas black dots represent non‐variable genes. (D) Principal component analysis showing the distribution of cells across different samples after quality control and normalization. (E) JackStraw analysis evaluating the statistical significance of principal components. (F) Elbow plot showing the standard deviation of each principal component. Based on the PCA, JackStraw analysis, and elbow plot, the top 20 principal components were selected for subsequent clustering and dimensionality reduction analyses. HVGs, highly variable genes; PCA, principal component analysis; PCs, principal components; UMI, unique molecular identifier.

**Supplementary Figure 2.** Validation of cell type annotation using marker gene expression. (A) t‐distributed stochastic neighbour embedding (t‐SNE) clustering of single‐cell RNA sequencing data, revealing the overall cellular landscape and distinct transcriptionally defined cell clusters. (B) Validation of cell type annotation based on canonical marker gene expression, demonstrating the correspondence between identified clusters and known cell type‐specific markers, confirming the accuracy of cell type assignment.

**Supplementary Table 1.** Non‐redundant interferon‐gamma response genes (IFNG‐RGs).

**Supplementary Table 2.** Primer sequences for RT‐qPCR validation.

**Supplementary Table 3.** GO enrichment of candidate genes.

**Supplementary Table 4.** KEGG pathway enrichment of candidate genes.

**Supplementary Table 5.** Immune cell correlation network metrics.

**Supplementary Table 6.** Key gene‐immune cell correlations.

**Supplementary Table 7.** Altered KEGG pathways in pSS microenvironment.

**Supplementary Tables 8–11.** Functional enrichment of individual key genes.

## Data Availability

The datasets analysed in this study are publicly available in the GEO repository (https://www.ncbi.nlm.nih.gov/geo/) under accession numbers GSE84844, GSE48378, and GSE157278. Additional data generated from RT‐qPCR experiments are available from the corresponding author upon reasonable request.
